# Editorial: The role of cellular subtypes in striatal activity

**DOI:** 10.3389/fncel.2026.2010201

**Published:** 2026-10-14

**Authors:** Elodie Fino, Maxime Assous, Marc Vincent Fuccillo

**Affiliations:** 1 Aix-Marseille University, INSERM U1249, INMED, Marseille, France; 2 School of Biosciences, Cardiff University, Cardiff, Wales, United Kingdom; 3 Department of Neuroscience, Perelman School of Medicine, University of Pennsylvania, Philadelphia, PA, United States

**Keywords:** microcircuits interconnection, neuromodulation, neuronal identity, striatum, subtypes diversity

The basal ganglia are central to the selection and execution of actions, integrating diverse information to shape motor, cognitive, and motivational behaviors. Within this network, the striatum occupies a key position where convergent cortical, thalamic, and neuromodulatory inputs are transformed into patterns of activity that ultimately influence basal ganglia output. While the functional organization of the striatum has long been framed around the opposing actions of direct- and indirect-pathway striatal projection neurons (SPNs), it is now increasingly clear that striatal function emerges from a far more complex interplay of neuronal populations. SPNs interact with diverse GABAergic and cholinergic interneurons, receive heterogeneous afferent inputs, and are continuously shaped by dopamine and other neuromodulatory systems. The resulting microcircuits are highly organized in space and function, and their cellular composition is likely to determine how information is integrated and transformed within the striatum. Understanding this complexity is therefore essential for linking cellular mechanisms to circuit activity and behavior. Recent advances in genetic targeting, transcriptomics, anatomical mapping and cell-type-specific recording and manipulation now make it possible to examine this diversity across molecular, cellular, circuit and behavioral scales.

The eight articles gathered in this Research Topic comprises *4 original articles, 2 reviews and 2 mini-reviews*. Together, these contributions illustrate the field's transition from relatively categorical descriptions of striatal cell types toward a multidimensional understanding of cellular identity, spatial organization, connectivity and function. A first theme emerging from this Research Topic is that neuronal identity is multidimensional. Electrophysiological classifications that have guided much of the *in vivo* literature, such as phasically active neurons (PAN), tonically active neurons (TAN), and fast-spiking interneurons (FSI), do not necessarily correspond in a straightforward manner to molecularly defined cell types. Apicella et al. revisit the classical PAN-TAN-FSI framework and emphasize both its usefulness and its limitations. This perspective is complemented by by Naon et al., who provide a detailed translatomic characterization of dorsal striatal PV interneurons, identifying a molecular signature that distinguishes them from their nucleus accumbens counterparts. Together, these studies highlight the importance of integrating molecular identity with electrophysiological properties and circuit function rather than considering any single feature as sufficient to define a neuronal subtype.

Cellular diversity is also strongly linked to anatomical organization. Muhsinov et al. provide a three-dimensional mapping of striatal SST-, PV-, and TH-expressing interneurons, revealing distinct spatial distributions across the mouse dorsal striatum. Such organization is likely to have important functional consequences because the position of a neuron determines the inputs it receives, the functional territory it belongs to and the local circuits it can influence. This spatial dimension is particularly evident in the review by Lemos et al. on striatal cholinergic interneurons. Although cholinergic interneurons constitute a relatively small population, their extensive influence on striatal networks is highly dependent on their regional location. Their properties and interactions with dopamine and other neuronal populations differ across striatal territories, emphasizing that even a genetically defined cell population may not represent a functionally homogeneous entity.

Another important highlight in this Research Topic further is that cellular diversity becomes meaningful through circuit interactions. Striatal activity results from the integration of convergent excitatory inputs and local inhibitory mechanisms, rather than from the activity of individual neuronal classes in isolation. The study by Karube et al., adds another organizational dimension by showing that pallidostriatal inhibitory projections preferentially target the matrix compartment, including SPNs and cholinergic interneurons. These findings illustrate how cell-type-specific interactions are embedded within the striosome-matrix architecture and suggest a route through which GPe inputs may shape compartment-specific striatal processing. Similarly, the review by Long and Masmanidis revisit the longstanding distinction between dopamine's roles in reinforcement learning and movement and advance a “learning primacy” hypothesis, arguing that understanding dopamine's behavioral functions requires resolving how dopaminergic signals act on specific striatal cellular and synaptic substrates across distinct timescales. These studies reinforce the view that the functional consequences of a given signal depend critically on the nature of the targeted cells, their location and how they are embedded within the larger network.

Finally, understanding cellular subtypes is essential for explaining how striatal circuits become dysfunctional in disease. Nelson et al. reports developmental alterations in SPN excitability in the Fmr1–/y mouse model, highlighting the importance of the temporal evolution of cellular dysfunction. Thabault and Galvan, in turn, review the contribution of PV interneurons to striatal dysfunction in Huntington's disease and autism spectrum disorder. Together, these studies illustrate that pathological changes cannot necessarily be described as a global increase or decrease in striatal activity. Different cell populations may be affected through distinct mechanisms and at different stages, potentially leading to complex and evolving alterations in circuit dynamics.

Taken together, this Research Topic emphasizes that cellular diversity is not simply a descriptive feature of the striatum; it is a fundamental determinant of its function. Molecular identity, developmental stage, electrophysiological properties, anatomical position, connectivity, and neuromodulatory sensitivity provide complementary dimensions through which neuronal populations contribute to striatal computation. This perspective places cellular heterogeneity at the center of our understanding of striatal circuits and underscores the need for an integrated view of cellular diversity, circuit organization, and behavior in the striatum.

